# Predicting delayed diagnosis in dementia: the impact of coping style

**DOI:** 10.1002/alz70858_096123

**Published:** 2025-12-23

**Authors:** Isaac Duncan‐Cross, Ben Hicks, Eleanor Miles

**Affiliations:** ^1^ University of Sussex, Brighton, East Sussex, United Kingdom; ^2^ Brighton and Sussex Medical School, Brighton, United Kingdom; ^3^ University of Sussex, Brighton, United Kingdom

## Abstract

**Background:**

Individuals with symptoms of dementia will often wait long periods before seeking a diagnosis, delaying access to support. Since coping styles can predict various health outcomes, including service use, this study explored whether individuals’ coping would predict diagnostic delays for dementia.

**Method:**

We assessed coping via the dispositional *Brief‐COPE* in 935 individuals recently diagnosed with dementia and 697 carers, all DETERMIND cohort members. Principal component analysis reduced coping strategies into principal factors, while hierarchical regression assessed the impact of these factors on diagnostic delay, controlling for participant demographics. Delay was measured temporally (self‐reported delay between symptom onset and diagnosis) and through symptom severity at diagnosis (Standardised Mini‐Mental State Examination, Clinical Dementia Rating).

**Result:**

Three principal factors were generated, representing ‘approach’, ‘avoidant’, and ‘support seeking’ coping styles. The coping styles of individuals with dementia predicted symptom severity, while temporal delays were predicted primarily by carers’ coping. Avoidant coping predicted more severe symptoms at diagnosis but shorter reported delays, whereas approach coping showed the opposite effect.

**Conclusion:**

Our findings suggest that understanding more about how individuals with dementia and their carers cope with stress may help further explain variation in diagnostic delays.

